# Confirmation of *Plasmodium falciparum* in vitro resistance to monodesethylamodiaquine and chloroquine in Dakar, Senegal, in 2015

**DOI:** 10.1186/s12936-017-1773-4

**Published:** 2017-03-16

**Authors:** Silman Diawara, Marylin Madamet, Mame Bou Kounta, Gora Lo, Khalifa Ababacar Wade, Aminata Nakoulima, Raymond Bercion, Rémy Amalvict, Mamadou Wague Gueye, Bécaye Fall, Bakary Diatta, Bruno Pradines

**Affiliations:** 1 Laboratoire d’étude de la Chimiosensibilité du Paludisme, Fédération des Laboratoires, Hôpital Principal de Dakar, Dakar, Senegal; 2 Unité Parasitologie et Entomologie, Département des Maladies Infectieuses, Institut de Recherche Biomédicale des Armées, Marseille, France; 3 Unité de Recherche sur les Maladies Infectieuses et Tropicales Emergentes, UM 63, CNRS 7278, IRD 198, Inserm 1095, Aix Marseille Université, Marseille, France; 4 Centre National de Référence du Paludisme, Marseille, France; 5 Service des Urgences, Hôpital Principal de Dakar, Dakar, Senegal; 6 Centre Medical Inter-armées, Dakar, Senegal; 7 Laboratoire de Bactériologie Virologie, Université Cheikh Anta Diop, CHU Le Dantec, Dakar, Senegal; 8 Service de Pédiatrie, Hôpital Principal de Dakar, Dakar, Senegal; 9 Laboratoire d’Analyses Médicales, Institut Pasteur, Dakar, Senegal; 10 Chefferie, Hôpital Principal de Dakar, Dakar, Senegal

**Keywords:** Malaria, *Plasmodium falciparum*, Anti-malarial drug, In vitro, Resistance, Senegal

## Abstract

**Background:**

In response to increasing resistance to anti-malarial drugs, Senegal adopted artemisinin-based combination therapy (ACT) as the first-line treatment for uncomplicated malaria in 2006. However, resistance of *Plasmodium falciparum* parasites to artemisinin derivatives, characterized by delayed parasite clearance after treatment with ACT or artesunate monotherapy, has recently emerged and rapidly spread in Southeast Asia. After 10 years of stability with rates ranging from 5.6 to 11.8%, the prevalence of parasites with reduced susceptibility in vitro to monodesethylamodiaquine, the active metabolite of an ACT partner drug, increased to 30.6% in 2014 in Dakar. Additionally, after a decrease of the in vitro chloroquine resistance in Dakar in 2009–2011, the prevalence of parasites that showed in vitro chloroquine resistance increased again to approximately 50% in Dakar since 2013. The aim of this study was to follow the evolution of the susceptibility to ACT partners and other anti-malarial drugs in 2015 in Dakar. An in vitro test is the only method currently available to provide an early indication of resistance to ACT partners.

**Results:**

Thirty-two *P. falciparum* isolates collected in 2015 in Dakar were analysed using a standard ex vivo assay based on an HRP2 ELISA. The prevalence of *P. falciparum* parasites with reduced susceptibility in vitro to monodesethylamodiaquine, chloroquine, mefloquine, doxycycline and quinine was 28.1, 46.9, 45.2, 31.2 and 9.7%, respectively. None of the parasites were resistant to lumefantrine, piperaquine, pyronaridine, dihydroartemisinin and artesunate. These results confirm an increase in the reduced susceptibility to monodesethylamodiaquine observed in 2014 in Dakar and the chloroquine resistance observed in 2013. The in vitro resistance seems to be established in Dakar. Additionally, the prevalence of parasites with reduced susceptibility to doxycycline has increased two-fold compared to 2014.

**Conclusions:**

The establishment of a reduced susceptibility to monodesethylamodiaquine as well as chloroquine resistance, and the emergence of a reduced susceptibility to doxycycline are disturbing. The in vitro and in vivo surveillance of anti-malarial drugs must be implemented in Senegal.

## Background

In response to increasing resistance to anti-malarial drugs, Senegal adopted an artemisinin-based combination therapy (ACT) as the first-line treatment for uncomplicated malaria in 2006 [1]. However, the resistance of *Plasmodium falciparum* parasites to artemisinin derivatives, characterized by delayed parasite clearance after treatment with ACT or artesunate monotherapy, has recently emerged and rapidly spread in Southeast Asia [2, 3]. No evidence exists for the emergence of artemisinin resistance in Senegal. However, active surveillance of temporal trends in the ex vivo susceptibility to the anti-malarial drugs used as partner drugs in ACT, such as monodesethylamodiaquine, the active metabolite of amodiaquine (artesunate–amodiaquine), lumefantrine (artemether–lumefantrine), piperaquine (dihydroartemisinin–piperaquine) or pyronaridine (artesunate–pyronaridine), is essential. After 10 years of stability with rates ranging from 5.6 to 11.8% [4–6], the prevalence of parasites with reduced susceptibility to monodesethylamodiaquine increased to 30.6% in 2014 in Dakar [7]. The prevalence of *P. falciparum* isolates with reduced susceptibility to lumefantrine remained low, below 3%, until 2014 [4–7]. Additionally, after a decrease in the in vitro chloroquine resistance in Dakar in 2009–2011 that paralleled the withdrawal of chloroquine treatment [4, 5], the prevalence of parasites with in vitro chloroquine resistance increased again to approximately 50% in Dakar since 2013 [6, 7]. The emergence and re-emergence of parasites with in vitro resistance to monodesethylamodiaquine and chloroquine is disturbing.

The aim of this study was to follow the evolution of the susceptibility to ACT partners and to other anti-malarial drugs in 2015 in Dakar.

## Methods

### Patients and sample collection

Thirty-two *P. falciparum* isolates were collected from September to November 2015 and analysed using the standard in vitro assay based on HRP2 ELISA at the Hôpital Principal of Dakar. Nineteen isolates came from patients (59.4%) who were recruited from the emergency unit. The remainder came from the Inter-armies Medical Centre of Dakar (21.9%), the Institut Pasteur of Dakar and other departments of the Hôpital Principal of Dakar.

One of the 32 patients was not from Dakar or its districts. Two patients had travelled during the previous month (one in Senegal and one in Saudi Arabia).

Anti-malarial treatment prior to admission was not recorded. At the Inter-armies Medical Centre of Dakar and the Institut Pasteur of Dakar, the patients were diagnosed only and were not treated. At the Hôpital Principal of Dakar, the patients were treated with diverse treatment protocols (i) quinine (9.5%), (ii) intra-venous artesunate followed by artemether–lumefantrine (4.8%), (iii) intra-venous artesunate in combination with doxycycline followed by artemether–lumefantrine–doxycycline (9.5%), or (iv) intra-muscular artemether with doxycycline followed by artemether–lumefantrine–doxycycline (76.2%). All of these patients were successfully cured.

Venous blood samples were collected in Vacutainer^®^ ACD tubes (Becton–Dickinson, Rutherford, NJ, USA) prior to patient treatment. Thin blood smears were stained using a RAL^®^ kit (Réactifs RAL, Paris, France) based on eosin and methylene blue and were examined to determine the level of *P. falciparum* parasitemia, which ranged from 0.066 to 2.46%. The evaluation of the ex vivo susceptibility to anti-malarial drugs was performed using the same venous blood samples used for diagnostic analysis.

### Drugs

Chloroquine, monodesethylamodiaquine, lumefantrine, piperaquine, pyronaridine, quinine, mefloquine, dihydroartemisinin, artesunate and doxycycline were distributed into 96-well plates as previously described [7]. The batches of the plates were validated on the chloroquine-resistant W2 strain (Indochina) (MR4, Virginia, USA). The clonality of this strain was regularly verified in our laboratory and by an independent laboratory from the Worldwide Antimalarial Resistance Network (WWARN) using PCR genotyping of the polymorphic genetic markers *msp1* and *msp2* and microsatellite loci [8, 9].

### Ex vivo assay

The susceptibility of *P. falciparum* was evaluated using the ex vivo assay based on HRP2 ELISA as previously described [7]. The threshold values for the reduced in vitro susceptibility or resistance were the following: 61, 115, 135, 60, 12, 12, 77, 611, 30, and 37 µM for monodesethylamodiaquine, lumefantrine, piperaquine, pyronaridine, dihydroartemisinin, artesunate, chloroquine, quinine, mefloquine and doxycycline, respectively [4, 10].

### Statistical analysis

The IC_50_ values were expressed as the geometric mean of the IC_50_ and a confidence interval of 95% (CI 95%) after logarithmic transformation. Cross-susceptibility was analysed using the Pearson correlation.

## Results

The average parameter estimates and the prevalence of isolates with ex vivo reduced susceptibility to the ten anti-malarial drugs evaluated were shown in Table 1.

**Table 1:** Ex vivo susceptibility to standard anti-malarial drugs of 32 *Plasmodium falciparum* isolates from Dakar collected from September 2014 to November 2015. The results were compared to the *P. falciparum* W2 clone tested under the same conditions

Drugs	Isolate IC_50_ mean [CI 95%]	W2 IC_50_ mean [CI 95%]	Resistance cut-off	% (no) of reduced susceptibility
Monodesethylamodiaquine	28.9 nM [21.3–39.0]	75 nM [70–80]	61 nM	28.1 (9/32)
Chloroquine	62.2 nM [35.0–110.6]	324 nM [199–351]	77 nM	46.9 (15/32)
Lumefantrine	3.5 nM [2.3–5.2]	12.4 nM [9.7–18.3]	115 nM	0 (0/32)
Mefloquine	26.0 nM [18.4–36.7]	18.3 nM [16.8–19.0]	30 nM	45.2 (14/31)
Quinine	220.6 nM [157.6–308.7]	294 nM [277–314]	611 nM	9.7 (3/31)
Piperaquine	36.5 nM [26.5–50.4]	31.5 nM [28.8–34.4]	135 nM	0 (0/30)
Pyronaridine	8.7 nM [5.8–13.2]	26.0 nM [21.5–25.7]	60 nM	0 (0/30)
Dihydroartemisinin	1.54 nM [0.99–2.39]	1.35 nM [1.14–1.60]	12 nM	0 (0/28)
Artesunate	2.13 nM [1.27–3.59]	1.63 nM [1.41–1.92]	12 nM	0 (0/28)
Doxycycline	25.7 µM [19.5–33.9]	11.3 µM [10.0–12.7]	37 µM	31.2 (10/32)

*CI 95%* confident interval 95%

The W2 clone IC_50_ means are the results of four independent experiments

The coefficient of correlation was 0.79 (p < 0.0001) for chloroquine/monodesethyamodiaquine, 0.32 (p = 0.085) for chloroquine/piperaquine and 0.34 (p = 0.062) for monodesethylamodiaquine/piperaquine.

## Discussion

In the absence of the strong validation of the association between a molecular marker and resistance to the components of ACT (lumefantrine, amodiaquine, piperaquine, pyronaridine), an in vitro test is the only method currently available to provide an early indication of a change in the resistance to ACT partner drugs. Temporal surveillance of the in vitro susceptibility from the same site using identical or comparative methodology is the most suitable method to detect emerging and spreading resistance. Additionally, an in vitro test is the only method to assess cross-resistance between antimalarial drugs.

In the absence of standardized in vitro and ex vivo tests, the validation of the batches of plates and of a method that uses a *P. falciparum* strain (regular controls of clonality) is essential.

These in vitro and ex vivo tests were the only approach to compare results from year to year, among sites or laboratories. The in vitro effects, the IC_50_ values and the threshold for in vitro resistance depend on the method used. The methodology (i.e., an isotopic test versus an immunoenzymatic test), gas conditions (i.e., O_2_ and CO_2_ and the incubation time are conditions that may affect the in vitro effects and the IC_50_ values [11–15].

The same methodology has been used in our studies since 2013. The results for the susceptibility of the W2 clone to the different anti-malarial drugs tested during the last 6 years allow a comparison of the year over year data.

The prevalence of *P. falciparum* parasites with reduced susceptibility to monodesethylamodiaquine was 28.1% in Dakar in 2015, which confirms the increase observed in 2014 (from 5.6% in 2013 to 30.6% in 2014) (p = 0.045 between 2013 and 2015) [6, 7]. The geometric means were comparable in 2014 and 2015 (25.3 and 28.9 nM, respectively, p = 0.517, Welch two sample *t* test). A three-fold increase was observed since 2013 (geometric mean, 9.8 nM [95% confidence interval, 5.5–17.4]) [6]. A reduced susceptibility to monodesethylamodiaquine seems to be established in Dakar since 2014. These data are consistent with previous data from Thies, which showed changes in the response to the drug amodiaquine. Specifically, the *P. falciparum* parasites became less susceptible to amodiaquine between 2008 and 2011 [16]. Susceptibility to monodesethylamodiaquine must be monitored in Senegal. Reduced susceptibility in vitro is an early warning of impeding resistance. The latest published clinical study on artesunate–amodiaquine in Senegal dated from 2012 [17]. Artesunate–amodiaquine was highly effective with an adequate clinical and parasitological response of 99.27% at day 42.

The persistence of the increase of reduced in vitro susceptibility to monodesethylamodiaquine could be explained by the use of artesunate–amodiaquine as the treatment for uncomplicated malaria. The use of this therapy could generate the emergence of parasites resistant to amodiaquine and to monodesethylamodiaquine [18]. Secondarily, cross-resistance (r = 0.79, p < 0.0001) between monodesethylamodiaquine and chloroquine may be the cause of the resistance or reduced susceptibility to both monodesethylamodiaquine and chloroquine [4, 19]. This phenomenon of cross-resistance could explain the increased in vitro resistance to chloroquine, which is no longer used in Senegal. However, an increase of chloroquine resistance was observed earlier than for monodesethylamodiaquine [6]. In fact, after a decrease of in vitro chloroquine resistance in Dakar in 2009–2011 that paralleled the withdrawal of chloroquine treatment [4, 5], the prevalence of parasites resistant in vitro to chloroquine increased again to approximately 50% in Dakar since 2013 [6, 7]. The results of 2015 confirmed this increase, which nevertheless remained stable (46.9%). This situation has not only been observed in Dakar. The in vitro susceptibility to chloroquine increased significantly between 2008 and 2011 in Thies [16] as well. Additionally, the prevalence of the mutation K76T in the *P. falciparum* chloroquine resistance transporter gene (*pfcrt*) increased again to 60% in 2006–2009 in Pikine (a suburb of Dakar) after a sharp decrease due to the cessation of chloroquine use [20].

The second ACT recommended for uncomplicated malaria treatment in Senegal is a combination of artemether–lumefantrine. In 2015, no isolate with reduced in vitro susceptibility to lumefantrine in Dakar was known. Since the introduction of ACT in Senegal, the prevalence of *P. falciparum* isolates with reduced susceptibility to lumefantrine remained low, below 3%, in Dakar [4–7]. Lumefantrine did not show significant cross-resistance with the other standard anti-malarial drugs [21], and this result is consistent with clinical outcomes. All the patients in this study, who were treated by artemether–lumefantrine–doxycycline after artesunate, artesunate–doxycycline or artemether–doxycycline, were successfully cured. The artemether–lumefantrine combination was highly effective with an adequate clinical and parasitological response of 99.2% at day 42 in suburbs of Dakar in 2010–2011 and 100% in 2012–2013 [17, 22].

The dihydroartemisinin–piperaquine combination has been recently introduced in Senegal as a second-line for the treatment of uncomplicated malaria. In 2015, no isolate with reduced in vitro susceptibility to piperaquine was observed in Dakar. The geometric means were comparable between 2013 and 2015 (32.2 nM in 2013, 34.5 in 2014 and 36.5 in 2015). Additionally, piperaquine did not show in vitro cross-resistance with chloroquine and monodesethylamodiaquine in isolates collected in Dakar (r = 0.32, p = 0.085 and r = 0.34, p = 0.062, respectively). The piperaquine in vitro susceptibility was not associated with the *pfcrt* K76T mutation involved in chloroquine resistance [23]. The dihydroartemisinin–piperaquine combination was highly effective, with an adequate clinical and parasitological response of 99.1% at day 42 in the suburbs of Dakar in 2010–2011 and 100% in 2012–2013 [17, 22].

Pyronaridine–artesunate, the most recent ACT, is currently under development for treatment of uncomplicated malaria. In 2015, no isolate with reduced in vitro susceptibility to pyronaridine was detected in Dakar. The geometric mean in 2015 in Dakar (8.72 nM) was intermediate between those observed in 2013 in Dakar (5.8 nM) or in Dielmo, in southeastern Senegal, in 1996 and 1997 (3.8 and 4.5 nM) and in 2014 in Dakar (10.5 nM) [6, 7, 24, 25]. However, a decrease in pyronaridine susceptibility is feared in the future due to the association of in vitro reduced pyronaridine susceptibility with the *pfcrt* K76T mutation [26]. No clinical data on artesunate–pyronaridine therapy are available from Senegal.

In 2015, no isolates with reduced in vitro susceptibility to dihydroartemisinin and to artesunate was found in Dakar. These results are consistent with those observed in previous studies in Dakar [4–6, 9]. A limitation of the present study was the absence of the determination of in vitro susceptibility to artemisinin derivatives by the ring survival test, which is a better indicator of in vitro artemisinin resistance. Clinical resistance to artemisinin was manifested by an increase in the ring-stage survival rate after contact with artemisinin [27–29].

Three *P. falciparum* isolates (9.7%) showed reduced in vitro susceptibility to quinine. This prevalence is slightly above that observed in Senegal for 15 years (2.8 to 6%) [4–7, 9, 30]. The two patients treated with quinine were successfully cured. Quinine is currently used less and less in Senegal in favour of intra-venous artesunate or an intra-muscular artemether injection.

Since 2009, the increase of the prevalence of parasites with reduced in vitro susceptibility to mefloquine has stabilized to approximately 50% [4–7]. Mefloquine is rarely, if ever, used in Senegal.

Data on the in vitro susceptibility to doxycycline are disturbing. Since 2010, the prevalence of *P. falciparum* isolates with reduced susceptibility to doxycycline has gradually increased (10.3% in 2010 and 16.7% in 2014), and it doubled in 2015 (31.2%) [5, 7]. Additionally, the geometric mean of the doxycycline IC_50_ in 2015 (25.7 µM) increased by a factor 2–3 in comparison with 2009, 2010 and 2014 (9.2, 11.6 and 8.5 µM, respectively) [4, 5, 7]. These changes must be monitored because doxycycline is used in combination with artesunate and artemether in the Hôpital Principal of Dakar.

## Conclusion

The establishment of reduced in vitro susceptibility to monodesethylamodiaquine and chloroquine resistance as well as the emergence of reduced susceptibility to doxycycline are disturbing. A limitation of this study was the low number of *P. falciparum* isolates tested. In vitro and in vivo surveillance of anti-malarial drugs must be implemented. The reduced in vitro susceptibility to monodesethylamodiaquine and doxycycline may quickly impact the clinical efficacy of the artemisinin combinations used in the Hôpital Principal of Dakar and in Senegal. In this context, only the artemisinin derivatives would be effective, similar to monotherapy. However, resistance of *P. falciparum* parasites to artemisinin derivatives, characterized by delayed parasite clearance after treatment with artesunate monotherapy or ACT, has recently emerged and rapidly spread in Southeast Asia, but has not had an impact up to now in Africa.

## Abbreviations

ACT: artemisinin-based combination therapy
CI 95%: 95% confidence interval
ELISA: enzyme-linked immunosorbent assay
HRP2: histidine rich protein II
IC_50_: inhibitory concentration 50%
PCR: polymerase chain reaction
*Pfcrt*: *Plasmodium falciparum* chloroquine resistance transporter gene
WWARN: Worldwide antimalarial resistance network
